# Type of sexual intercourse experience and suicidal ideation, plans, and attempts among youths: a cross-sectional study in South Korea

**DOI:** 10.1186/s12889-016-3895-y

**Published:** 2016-12-07

**Authors:** Geum Hee Kim, Hyeong Sik Ahn, Hyun Jung Kim

**Affiliations:** 1 Department of School Health Education, Sanggye High School, 432, Nohaero, Nowon-gu, Seoul 01761 Korea; 2 Department of Preventive Medicine, College of Medicine, Korea University, 73, Inchon-ro, Seongbuk-gu, Seoul 02841 Korea

**Keywords:** Lesbian, Gay, Bisexual, Youths, Sexual intercourse, Suicide, Violence, Sexually transmitted diseases

## Abstract

**Background:**

Despite abundant theoretical evidence of higher rates of suicide among lesbian, gay, and bisexual (LGB) youths, little is known about the relationship between suicide and types of sexual intercourse experience in youths. This study examines the association between the type of intercourse experience and suicide risk outcomes (SROs: suicidal ideation, plans for suicide, suicidal attempts) from the Korea Youth Risk Behavior Web-based Survey.

**Methods:**

We analyzed cross-sectional data from 146,621 students aged 12–17 years for the years 2012 and 2013. We defined lesbian, gay, or bisexual youth as youths who engaged in a type of sexual intercourse (same-sex or both-sex intercourse). A chi-square test and logistic regression analysis were used to evaluate the association between intercourse experience and SROs.

**Results:**

The results showed that the prevalence of suicidal ideation was higher among youths with same-sex intercourse experience (45.9% for females, 33.7% for males) than among youths with opposite-sex intercourse experience (42.2% for females, 23.8% for males) and those with no experience in intercourse (21.0% for females, 12.7% for males). After adjusting for revealed risk factors that were associated with suicide risks, among males, suicide risks based on intercourse experience seemed to increase in the following order: no experience in sexual intercourse, opposite-sex, same-sex, and then both-sexes sexual intercourse experience. Same- and both-sexes intercourse related SROs are strongly linked to violence (being physically assaulted, threatened, or bullied) and sexually transmitted diseases (STDs), including HIV infection. Those having no sexual intercourse experience showed the least probability of suicide risks among youths.

**Conclusion:**

The SROs of youths with same-sex or both-sex intercourse experience had strong associations with gender (males), violence, and STDs. Therefore, school educators must continue to advocate for and to implement LGB inclusive policies and programs in order to promote safe and supportive learning environments where all students are protected from health risk behaviors.

## Background

Suicide is the leading cause of mortality among Korean youths aged 10–19 years, which demonstrates that suicide among youths is a significant issue [1]. Suicidal behavior includes suicidal ideation, plans, attempts, and death through a medically serious action [2]. Variables that influence the suicidal behavior were depression and ever having had sexual intercourse as youths [2]. Media reports convey the message that lesbian, gay, or bisexual (LGB) youths have much greater rates of suicide than non-LGB youths [3]. LGB youths experience stress and depression during the formation of their sexual identity. They have been reported to demonstrate suicidal attempts 2–3 times more often than heterosexual youths [4, 5]. The prevalence of suicide in youths who engaged in a type of sexual intercourse (having had intercourse with the opposite-sex, same-sex, or both-sex intercourse) has been known to differ among races and countries [3]. To date, most studies on the outcomes for the risk of suicide as a result of sexual intercourse experience have been reported in western countries; very few studies have been done in Asian countries [6–8]. Although there have been several small studies elucidating the association between homosexuality and suicide, there have been no studies on the prevalence of the type of sexual intercourse or suicidal behavior by sexual intercourse experience in South Korean youths [8]. Therefore, we investigated the status of youths by type of sexual intercourse among all middle and high school students in South Korea. We assessed the direct effects of suicide risk outcomes (SROs: suicidal ideation, plans for suicide, suicidal attempts) due to type of sexual intercourse experience while controlling for other confounders known to affect SROs among youths. The objective of this study was to analyze and interpret the association between the prevalence of youths with sexual intercourse experience and SROs, by using the Korea Youth Risk Behavior Web-based Survey (KYRBS) database from the years 2012 and 2013.

## Methods

### Study design and population

This was a cross-sectional study using Korea’s representative Youth Risk Behavior Web-based Survey (KYRBS) database from 2012 and 2013. The study consisted of 146,621 students (from grades 7 to 12, aged 12 to 17 years) who were sampled randomly. Sampling for the KYRBS consisted of stratified randomization. The population consisted of stratified parameters, such as region and grade level. As for strata of variables, the number of sampled schools was distributed based on city, county, size of the city, and school type using a proportional allocation method to match population and sample compositions. At the stage of sample extraction, we used a stratified 2-stage cluster sample design, with the unit for primary extraction being the school and the unit for secondary extraction being the intact classes [9].

### Measures

KYRBS is a school-based, self-report from the Ministry of Education, Ministry of Health and Welfare, and the Korea Centers for Disease Control and Prevention (KCDC) to understand health behaviors among youths. The KYRBS has 126 questions that assess 15 domains of health risk behavior, such as smoking, drinking, obesity, sexual intercourse, suicidal behavior, and violence.

#### Type of sexual intercourse

This was measured by the question: “During your life, with whom have you had sexual intercourse experience?” Response options included I have never had sexual intercourse, opposite-sex, and same-sex intercourse experience. We classified students as having had intercourse with the opposite-sex, intercourse with the same-sex, intercourse with the both-sex, no experience in intercourse (no contact).

#### Suicide risk outcomes (SROs)

We measured three dichotomous questions: seriously considered suicide ideation, made a plan for suicide plans, and attempted suicide at least once during the past 12 months prior to the survey.

#### Control variables

We selected risk factors associated with type of sexual intercourse or SROs based on current literature [2, 5, 10–15]. We included the following risk factors occurring during the past 12 months prior to the survey. To measure violence, we used the question: “Have you had any experience of receiving treatment at a hospital for violence (being physically assaulted, threatened, or bullied) from friends, superiors, or adults?” We measured six socioeconomic-related factors; gender, grade, type of school, region, self-reported academic achievement, and perceived household economic status. We measured family structure by asking one question; living with both parents (both parents), living with mother (mother only), living with father (father only), or not living with parents (neither parent). We included questions about two different health risk behaviors. To calculate smoking habits, the question: “During the past 30 days prior to the survey, have you smoked at least one cigarette?” was used. To determine the effect of drinking, we included the following question: “During the past 12 months prior to the survey, have you had at least one alcoholic drink alone?” We also included a variable to assess the evidence of an association with suicidal behavior: subjective perception of body habits, which is perceiving oneself as being very obese as opposed to being overweight, underweight, or normal (perceived obesity). Responses for subjective perception of health status were quite poor or poor as opposed to excellent, very good, or good (perceived health status). Finally, sexual health risk behavior was assessed with a single measure (“Have you ever been diagnosed with sexually transmitted diseases (STDs) including HIV infection from sexual intercourse?”).

### Statistical analysis

Using IBM SPSS Statistics Version 20, we conducted statistical analysis on weighted data in order to interpret the data in a way that represented all students in grades 7–12 who were attending public schools in South Korea in 2012 and 2013. We merged the years of available data from 2012 and 2013 from the KYRBS.

We performed all statistical analyses on weighted data to adjust for subjects’ nonresponses and to account for the complex sampling design of the KYRBS. We compared the distribution of demographic characteristics (Table 1) and SROs (Table 2) by sexual intercourse experience using a chi-square test. The demographics of the subjects were shown as an unweighted sample size and a weighted percentage using composite sample descriptive statistics.

**Table 1:** Demographic characteristics by gender and the type of sexual intercourse experience: KYRBS

Merged 2012 ~ 2013 *n* = 146,621		Type of sexual intercourse, no. (%)^‡^							
		Females, *n* = 71,745				Males, *n* = 74,876			
Characteristics		No Contact	Opposite	Same	Both	No Contact	Opposite	Same	Both
Socioeconomic factors									
Gender									
Female		69,706 (97.2)	1,649 (2.2)	203 (0.3)	187 (0.3)				
Male						69,936 (93.3)	4,060 (5.5)	441 (0.6)	439 (0.6)
School type									
Single sex		24,326 (98.0)	423 (1.8)	41 (0.1)	26 (0.1)	22,975 (93.7)	1,213^†^ (5.2)	136† (0.5)	148^†^ (0.6)
Coeducation		45,380 (96.7)	1,226 (2.5)	162 (0.4)	161 (0.4)	46,961 (93.2)	2,847^†^ (5.6)	305† (0.6)	291^†^ (0.6)
Grade	Age								
7		11,513 (97.5)	200 (1.8)	31 (0.3)	42 (0.4)	12,306 (96.3)	345 (2.8)	54 (0.4)	70 (0.5)
8		11,646 (98.4)	136 (1.1)	35 (0.3)	25 (0.2)	12,224 (96.5)	293 (2.3)	72 (0.6)	66 (0.5)
9	≤ 14 years	11,771 (98.0)	161 (1.3)	32 (0.3)	31 (0.3)	12,278 (96.0)	341 (2.7)	75 (0.6)	80 (0.7)
10		11,484 (97.6)	238 (1.9)	24 (0.2)	29 (0.3)	11,946 (94.1)	610 (4.7)	72 (0.6)	76 (0.6)
11		11,897 (96.4)	400 (3.0)	38 (0.3)	29 (0.3)	10,659 (90.5)	998 (8.1)	80 (0.7)	79 (0.7)
12	≤ 17 years	11,395 (95.2)	514 (4.2)	43 (0.3)	31 (0.3)	10,523 (87.0)	1,473 (11.8)	88 (0.6)	68 (0.6)
Achivement									
High		6,577 (96.7)	112 (1.6)	38 (0.7)	54 (1.0)	8,491 (93.2)	404 (4.6)	77 (0.9)	109 (1.3)
Mid-high		16,997 (98.0)	309 (1.7)	34 (0.2)	22 (0.2)	16,507 (95.3)	645 (3.9)	61 (0.4)	80 (0.5)
Middle		19,716 (97.9)	373 (1.8)	38 (0.2)	29 (0.1)	18,702 (94.3)	965 (4.8)	102 (0.5)	77 (0.4)
Mid-low		18,087 (97.0)	489 (2.6)	46 (0.2)	31 (0.2)	17,082 (92.8)	1,109 (6.1)	113 (0.6)	91 (0.5)
Low		8,329 (94.8)	366 (4.0)	47 (0.6)	51 (0.6)	9,154 (89.1)	937 (9.2)	88 (0.9)	82 (0.8)
Economic									
High		3,282 (94.0)	94 (2.8)	34 (1.3)	58 (1.9)	5,856 (89.4)	439 (7.7)	76 (1.1)	112 (1.8)
Mid-high		15,843 (97.7)	304 (1.9)	44 (0.3)	23 (0.2)	17,801 (94.6)	831 (4.5)	103 (0.5)	75 (0.4)
Middle		35,054 (98.0)	654 (1.7)	57 (0.1)	51 (0.2)	31,754 (94.6)	1,526 (4.5)	135 (0.4)	147 (0.5)
Mid-low		12,457 (96.6)	390 (2.9)	34 (0.3)	23 (0.2)	11,215 (92.6)	784 (6.5)	69 (0.5)	47 (0.4)
Low		3,070 (91.7)	207 (6.1)	34 (1.1)	32 (1.1)	3,310 (85.3)	426 (11.5)	58 (1.7)	58 (1.6)
Family structure									
Both parents		56,894 (97.8)	1,090 (1.8)	99 (0.2)	107 (0.2)	57,563 (94.2)	2,919 (4.8)	278 (0.5)	305 (0.5)
Mother only		7,350 (96.1)	264 (3.3)	31 (0.4)	15 (0.2)	6,204 (91.1)	504 (7.5)	57 (0.8)	35 (0.6)
Father only		3,434 (94.9)	163 (4.3)	15 (0.4)	9 (0.4)	3,967 (89.9)	356 (8.5)	35 (0.9)	30 (0.8)
Neither parent		2,028 (86.6)	132 (6.4)	58 (3.3)	56 (3.7)	2,202 (81.9)	281 (11.6)	71 (3.3)	69 (3.2)
Health risk behaviors									
Smoking									
No		66,577 (98.1)	1,105 (1.6)	104 (0.2)	100 (0.2)	60,994 (96.3)	1,772 (2.9)	262 (0.4)	251 (0.4)
Yes		3,129 (80.7)	544 (13.7)	99 (2.8)	87 (2.8)	8,942 (77.0)	2,288 (19.8)	179 (1.6)	188 (1.6)
Drinking									
No		67,533 (97.7)	1,330 (1.9)	158 (0.2)	123 (0.2)	66,739 (94.8)	2,903 (4.2)	359 (0.5)	353 (0.5)
Yes		2,173 (82.8)	319 (12.2)	45 (2.0)	64 (3.1)	3,197 (70.2)	1,157 (25.8)	82 (2.0)	86 (1.9)
Obesity^a^									
No		65,060 (97.2)	1,524 (2.2)	188 (0.3)	166 (0.3)	66,387 (93.3)	3,904 (5.5)	407 (0.6)	417 (0.6)
Yes		4,646 (96.7)	125 (2.5)	15 (0.3)	21 (0.5)	3,549 (94.4)	156 (4.1)	34 (0.8)	22 (0.7)
Health status^b^									
Good		64,047 (97.4)	1,391 (2.0)	178 (0.3)	162 (0.3)	66,317 (93.5)	3,752 (5.3)	398 (0.6)	405 (0.6)
Poor		5,659 (94.9)	258 (4.2)	25 (0.4)	25 (0.6)	3,619 (90.4)	308 (7.6)	43 (1.1)	34 (1.0)
STD diagnosis^c^									
No		69,706 (97.5)	1,570 (2.1)	134 (0.2)	118 (0.2)	69,936 (93.8)	3,863 (5.2)	338 (0.4)	349 (0.5)
Yes			79 (33.5)	69 (32.5)	69 (34.0)		197 (48.2)	103 (27.5)	90 (24.3)
Violence^d^									
No		68,529 (97.6)	1,498 (2.1)	111 (0.2)	107 (0.2)	67,156 (94.1)	3,591 (5.1)	307 (0.4)	302 (0.4)
Yes		1,177 (76.6)	151 (10.0)	92 (6.8)	80 (6.6)	2,780 (78.5)	469 (13.4)	134 (4.1)	137 (4.0)

Notes: All the variables presented *P* < 0.001, using a chi-square test, except ^†^ *P* > 0.05

^‡^Unweighted sample size and weighted percentage

^a^Perceived very obesity

^b^Responses for subjective perception of health status

^c^Diagnosed with sexually transmitted diseases (STDs) including HIV Infection from sexual intercourse

^d^Victim of violence (being physically assaulted, threatened, or bullied)

**Table 2:** Prevalence of suicide risk outcomes by the type of sexual intercourse experience: KYRBS

Merged 2012 ~ 2013 *n* = 146,621		Suicide risk outcomes, Responses ‘Yes’ = No. (%)^‡^					
		Females, *n* = 71,745			Males, *n* = 74,876		
Characteristics		Suicide ideation	Suicide plan	Suicide attempt	Suicide ideation	Suicide plan	Suicide attempt
Yes = No. (%)^‡^		15,603 (21.7)	5,240 (7.2)	3,912 (5.5)	10,102 (13.6)	3,637 (4.8)	2,127 (2.8)
Sexual intercourse							
No contact		14,726 (21.0)	4,787 (6.8)	3,494 (5.0)	8,838 (12.7)	3,003 (4.3)	1,678 (2.4)
Opposite sex		694 (42.2)	308 (18.7)	297 (18.0)	973 (23.8)	446 (10.9)	306 (7.4)
Same sex		92 (45.9)	74 (34.3)	57 (28.4)	147 (33.7)	93 (22.0)	73 (17.0)
Both sexes		91 (49.4)	71 (41.2)	64 (34.1)	144 (34.2)	95 (21.6)	70 (16.8)
Socioeconomic factors							
School type							
Single sex		5,052 (20.3)	1,550 (6.2)	1,084 (4.4)	3,219^†^ (13.2)	1,063 (4.3)	587 (2.4)
Coeducation		10,551 (22.4)	3,690 (7.8)	2,828 (6.1)	6,883^†^ (13.8)	2,574 (5.1)	1,540 (3.0)
Grade	Age						
7		2,615 (22.4)	1,049 (9.0)	823 (7.3)	1,553 (12.6)	611 (5.0)	328 (2.6)
8		2,852 (24.2)	1,128 (9.5)	849 (7.4)	1,710 (13.7)	704 (5.5)	427 (3.4)
9	≤ 12 years	2,698 (22.3)	975 (8.1)	714 (6.0)	1,804 (14.4)	679 (5.5)	408 (3.2)
10		2,502 (21.1)	719 (6.1)	576 (4.8)	1,742 (13.3)	600 (4.5)	386 (2.9)
11		2,685 (21.4)	802 (6.4)	553 (4.3)	1,689 (14.1)	545 (4.5)	295 (2.5)
12	≤ 17 years	2,251 (18.7)	567 (4.6)	397 (3.3)	1,604 (13.3)	498 (4.1)	283 (2.3)
Achivement							
High		1,235 (17.9)	391 (5.8)	258 (4.0)	1,109 (12.9)	441 (5.0)	265 (3.0)
Mid-high		3,239 (18.7)	1,058 (6.2)	693 (4.1)	2,038 (11.8)	712 (4.0)	404 (2.3)
Middle		3,760 (18.6)	1,155 (5.6)	831 (4.1)	2,439 (12.3)	821 (4.1)	438 (2.2)
Mid-low		4,586 (24.4)	1,544 (8.2)	1,208 (6.4)	2,655 (14.8)	924 (5.1)	500 (2.6)
Low		2,783 (32.0)	1,092 (12.4)	922 (10.5)	1,861 (18.2)	739 (7.3)	520 (5.1)
Economic							
High		734 (21.3)	310 (9.3)	226 (7.0)	945 (14.5)	467 (7.3)	308 (4.7)
Mid-high		3,069 (19.0)	1,033 (6.3)	759 (4.7)	2,223 (12.0)	771 (4.2)	425 (2.2)
Middle		6,937 (19.2)	2,227 (6.1)	1,648 (4.6)	3,894 (11.7)	1,329 (3.9)	737 (2.2)
Mid-low		3,533 (27.5)	1,115 (8.6)	841 (6.5)	2,053 (17.0)	619 (5.0)	368 (2.9)
Low		1,330 (40.3)	555 (17.3)	438 (13.6)	987 (25.9)	451 (11.8)	289 (7.5)
Family structure							
Both parents		11,771 (20.1)	3,751 (6.4)	2,764 (4.8)	7,788 (12.9)	2,733 (4.5)	1,535 (2.5)
Mother only		2,020 (26.7)	738 (9.9)	543 (7.3)	1,031 (15.1)	362 (5.2)	225 (3.2)
Father only		1,051 (29.4)	401 (11.0)	325 (9.1)	739 (16.8)	278 (6.2)	167 (3.6)
Neither parent		761 (35.6)	350 (17.0)	280 (13.9)	544 (22.0)	264 (11.1)	200 (8.4)
Health risk behaviors							
Smoking							
No		13,750 (20.2)	4,366 (6.4)	3,114 (4.6)	7,509 (12.0)	2,579 (4.1)	1,370 (2.2)
Yes		1,853 (47.9)	874 (23.1)	798 (20.9)	2,593 (22.4)	1,058 (9.1)	757 (6.4)
Drinking							
No		14,329 (20.7)	4,598 (6.6)	3,347 (4.9)	8,798 (12.6)	3,053 (4.3)	1,701 (2.4)
Yes		1,274 (49.3)	642 (25.2)	565 (21.8)	1,304 (29.0)	584 (13.2)	426 (9.7)
Obesity^a^							
No		14,162 (21.1)	4,684 (6.9)	3,520 (5.3)	9,408 (13.3)	3,357 (4.7)	1,958 (2.7)
Yes		1,441 (30.1)	556 (11.5)	392 (8.3)	694 (18.8)	280 (7.5)	169 (4.4)
Health status^b^							
Good		13,162 (19.9)	4,246 (6.4)	3,178 (4.9)	8,902 (12.6)	3,137 (4.4)	1,816 (2.5)
Poor		2,441 (40.7)	994 (16.9)	734 (12.5)	1,200 (30.9)	500 (13.1)	311 (7.9)
STD diagnosis^c^							
No		15,476 (21.5)	5,147 (7.1)	3,824 (5.4)	9,935 (13.4)	3,511 (4.7)	2,032 (2.7)
Yes		127 (61.2)	93 (44.4)	88 (42.8)	167 (44.6)	126 (34.5)	95 (24.7)
Violence^d^							
No		14,765 (20.9)	4,748 (6.7)	3,481 (5.0)	8,877 (12.5)	2,897 (4.0)	1,593 (2.2)
Yes		838 (55.3)	492 (32.8)	431 (28.7)	1,225 (35.1)	740 (21.6)	534 (15.6)

Notes: All the variables presented *P* ≤ 0.001, using a chi-square test, except ^†^ *P* > 0.05

^‡^Unweighted sample size and weighted percentage

Outcome variable (suicide risk outcomes) = binary variable Yes or No

^a^Perceived very obesity

^b^Responses for subjective perception of health status

^c^Diagnosed with sexually transmitted diseases (STDs) including HIV Infection from sexual intercourse

^d^Victim of violence (being physically assaulted, threatened, or bullied)

We used simple logistic regression analysis to examine the associations between SROs and type of sexual intercourse experience, along with all control variables. After which, we performed a multiple logistic regression analyses in order to predict SROs after controlling for family structure, health risk behaviors (smoking, drinking, perceived obesity, and perceived health status), STDs, and experiences of violence [15]. We were presented unadjusted and adjusted odds ratios (ORs) and 95% confidence intervals (CIs) by gender (Table 3).

**Table 3:** Associations between the type of sexual intercourse and suicide risk outcomes: KYRBS

Merged 2012 ~ 2013 *n* = 146,621^‡^	Suicide ideation, OR (95% CIs)		Suicide plan, OR (95% CIs)		Suicide attempt, OR (95% CIs)	
	Unadjusted	Adjusted	Unadjusted	Adjusted	Unadjusted	Adjusted
Female						
Sexual intercourse						
Opposite sex	.00	1.00	1.00	1.00	1.00	1.00
Same sex	1.16^†^ (0.88–1.53)	0.59* (0.42–0.83)	2.27 (1.66–3.09)	1.19^†^ (0.82–1.73)	1.81 (1.29–2.52)	0.76^†^ (0.52–1.12)
Both sexes	1.33^†^ (0.98–1.81)	0.63* (0.43–0.92)	3.06 (2.18–4.28)	1.50^†^ (0.99–2.26)	2.36 (1.67–3.33)	0.88^†^ (0.53–1.45)
No contact	0.36 (0.33–0.40)	0.62 (0.55–0.70)	0.31 (0.36–0.41)	0.66 (0.56–0.77)	0.24 (0.30–0.36)	0.54 (0.46–0.64)
Family structure						
Both parents	1.00	1.00	1.00	1.00	1.00	1.00
Mother only	1.44 (1.37–1.51)	1.28 (1.22–1.36)	1.60 (1.48–1.73)	1.38 (1.27–1.50)	1.57* (1.43–1.71)	1.32 (1.20–1.45)
Father only	1.65 (1.53–1.78)	1.39 (1.29–1.51)	1.82 (1.64–2.02)	1.44 (1.29–1.61)	2.00 (1.78–2.25)	1.54 (1.35–1.75)
Neither parent	2.19 (2.00–2.40)	1.47 (1.33–1.64)	3.01 (2.67–3.39)	1.55 (1.34–1.79)	3.20 (2.80–3.67)	1.47 (1.24–1.73)
Health risk behavior						
Smoking	3.62 (3.39–3.85)	2.34 (2.18–2.51)	4.41 (4.07–4.79)	2.34 (2.11–2.59)	5.45 (5.00–5.95)	2.77 (2.50–3.09)
Drinking	3.73 (3.46–4.02)	2.20 (2.01–2.40)	4.78 (4.37–5.24)	2.39 (2.13–2.67)	5.42 (4.92–5.96)	2.41 (2.14–2.71)
Obesity^a^	1.61 (1.51–1.71)	1.43 (1.34–1.53)	1.74 (1.59–1.91)	1.48 (1.34–1.64)	1.61 (1.45–1.78)	1.35 (1.21–1.52)
Health status^b^	2.74 (2.60–2.89)	2.42 (2.29–2.55)	2.97 (2.77–3.19)	2.45 (2.27–2.65)	2.78 (2.57–3.01)	2.23 (2.04–2.43)
STD diagnosis^c^	5.74 (4.42–7.44)	0.94^†^ (0.66–1.34)	10.43 (7.88–13.79)	0.81^†^ (0.56–1.17)	13.22 (9.83–17.79)	1.06^†^ (0.71–1.58)
Violence^d^	4.66 (4.22–5.14)	3.17 (2.83–3.56)	6.82 (6.11–7.61)	3.87 (3.39–4.43)	7.65 (6.82–8.59)	4.10 (3.55–4.72)
Male						
Sexual intercourse						
Opposite sex	1.00	1.00	1.00	1.00	1.00	1.00
Same sex	1.62 (1.31–2.01)	1.29* (1.01–1.64)	2.30 (1.80–2.93)	1.45* (1.10–1.92)	2.58 (1.95–3.40)	1.69* (1.22–2.35)
Both sexes	1.66 (1.35–2.03)	1.43 (1.16–1.78)	2.23 (1.74–2.88)	1.57* (1.19–2.07)	2.54 (1.92–3.37)	1.84 (1.32–2.56)
No contact	0.36 (0.33–0.40)	0.76 (0.70–0.83)	0.36 (0.32–0.40)	0.67 (0.60–0.76)	0.30 (0.26–0.34)	0.67 (0.58–0.78)
Family structure						
Both parents	1.00	1.00	1.00	1.00	1.00	1.00
Mother only	1.19 (1.11–1.28)	1.08 (1.00–1.16)	1.16 (1.04–1.29)	1.02^†^ (0.90–1.14)	1.30 (1.12–1.50)	1.10^†^ (0.94–1.28)
Father only	1.36 (1.25–1.48)	1.16 (1.06–1.26)	1.39 (1.23–1.58)	1.11^†^ (0.97–1.27)	1.45 (1.23–1.71)	1.08^†^ (0.91–1.30)
Neither parent	1.90 (1.72–2.09)	1.27 (1.15–1.42)	2.66 (2.32–3.03)	1.37 (1.19–1.58)	3.55 (3.04–4.14)	1.61 (1.32–1.92)
Health risk behavior						
Smoking	2.11 (2.01–2.23)	1.56 (1.47–1.66)	2.35 (2.19–2.53)	1.49 (1.36–1.63)	3.11 (2.84–3.41)	1.81 (1.62–2.02)
Drinking	2.83 (2.64–3.02)	1.82 (1.68–1.96)	3.38 (3.06–3.73)	1.94 (1.72–2.18)	4.41 (3.97–4.89)	2.19 (1.92–2.50)
Obesity^a^	1.51 (1.39–1.64)	1.24 (1.13–1.35)	1.64 (1.45–1.86)	1.26 (1.10–1.44)	1.64 (1.40–1.93)	1.22* (1.03–1.46)
Health status^b^	3.08 (2.88–3.30)	2.74 (2.56–2.95)	3.28 (2.96–3.63)	2.73 (2.44–3.05)	3.30 (2.91–3.74)	2.56 (2.23–2.93)
STD diagnosis^c^	5.19 (4.32–6.25)	1.35* (1.10–1.66)	10.71 (8.69–13.20)	1.80 (1.40–2.32)	11.86 (9.47–14.84)	1.34* (1.02–1.76)
Violence^d^	3.78 (3.53–4.04)	3.02 (2.81–3.24)	6.55 (6.01–7.13)	4.77 (4.35–5.23)	8.22 (7.40–9.14)	5.56 (4.97–6.22)

Notes: All the variables presented *P* ≤ 0.001, using simple and multiple logistic regression analysis, except *P < 0.05; ^†^ *P* > 0.05

OR = odds ratio; CIs = Confidence Intervals

Outcome variable: Suicide risk outcomes = Yes (Reference group; Suicide risk outcomes = No)

^‡^Unweighted sample size

^a^Perceived very obesity

^b^Perception of health status were quite poor or poor

^c^Diagnosed with sexually transmitted diseases (STDs) including HIV Infection from sexual intercourse

^d^Victim of violence (being physically assaulted, threatened, or bullied)

## Results

### The prevalence of type of sexual intercourse in youths

Table 1 demonstrates the prevalence of sexual intercourse in youths based on demographics. Among the 146,621 students (from grades 7 to 12), 3.9% had opposite-sex intercourse (2.2% for females, 5.5% for males), 0.5% had same-sex intercourse (0.3% for females, 0.6% males), and 0.5% had intercourse with both sexes (0.3% for females, 0.6% for males).

### The prevalence of SROs in youths

Table 2 presents the prevalence of SROs. The prevalence was higher among youths with same-sex intercourse experience for suicidal ideation (45.9% for females, 33.7% for males), plans for suicide (34.3% for females, 22.0% for males), and suicidal attempts (28.4% for females, 17.0% for males) than among youths with opposite-sex intercourse experience (*p <* 0.001). The prevalence was higher among youths who had STDs for suicidal ideation (61.2% for females, 44.6% for males), plans for suicide (44.4% for females, 34.5% for males), and suicidal attempts (42.8% for females, 24.7% for males) than for youths who did not have STDs. Additionally, the prevalence for suicidal ideation was higher among youths who were victims of violence (55.3% for females, 35.1% for males) than for youths who had not been victims of violence (p < 0.001). In our study, SROs based on sexual intercourse experience seemed to increase in the following order: no experience in intercourse, opposite-sex intercourse, same-sex intercourse, and then both-sexes intercourse experience (Table 2).

### The associations between type of sexual intercourse and SROs

In unadjusted analyses examining lesbian, gay, or bisexual youth defined by type of sexual intercourse, except for suicide ideation among females, we found that most youths with same-sex intercourse and both-sex intercourse compared with opposite-sex intercourse had increased odds of all SROs, with the odds ratios (ORs) ranging from 2.27 (95% confidence interval [CI] = 1.66–3.09) for plans of suicide among females with same-sex intercourse to 2.58 (95% CI = 1.95–3.40) for suicidal attempts among males with same-sex intercourse (all *p* < 0.001). All youths who had STDs increased the odds of all SROs compared to youths who had not been diagnosed with STDs (Table 2).

In the adjusted analyses, we added all the control variables (family structure, health risk behaviors, STDs including HIV infection, and experiences of violence) simultaneously. Compared to males with opposite-sex intercourse experience only, males who had experienced same-sex or both-sex intercourse differed significantly in their odds of SROs. On the other hand, among females, we found that females with same-sex and both-sex intercourse experience, compared to those with opposite-sex intercourse, had reduced odds of suicide ideation. In addition, females with same-sex and both-sex intercourse experience did not differ in suicide planning and attempts when compared to females with opposite-sex intercourse only. Males with same-sex intercourse experience were higher in suicidal ideation (adjusted odds ratio [AOR] = 1.29; 95% CI = 1.01–1.64), plans for suicide (AOR = 1.45; 95% CI = 1.10–1.92), and suicidal attempts (AOR = 1.69; 95% CI = 1.22–2.35) compared with males who had only experienced opposite-sex intercourse. Males suffering from STDs, including HIV infection, were higher in suicidal ideation (AOR = 1.35; 95% CI = 1.10–1.66), plans for suicide (AOR = 1.80; 95% CI = 1.40–2.32), and suicidal attempts (AOR = 1.34; 95% CI = 1.02–1.76) compared to males who did not have an STD. SROs were higher among youths who had been victims of violence (all AOR range = 3–5 for SROs) compared to youths who had not been victims of violence. When adjusted for the effects of the control variables (family structure, health risk behaviors, STDs, and violence), each SRO among youths with same- and both-sex intercourse tended to decrease. In particular, SROs among youths with same-sex and both-sex intercourse experience seemed to be strongly associated with STDs and violence. Our study demonstrated that after adjusting for the effect of STDs, violence, and associated factors, males with same-sex or both-sex intercourse experience had increased odds of all SROs when compared with opposite-sex intercourse only. However, females with same-sex and both-sex intercourse experience did not differ significantly in suicide planning and attempts. We detected that violence had strong associations with SROs of youths who had experienced same-sex or both-sex intercourse, with STDs including HIV infection (Table 3).

## Discussion

We investigated the effects of the type of sexual intercourse among South Korean youths on suicide risk outcomes using Korea’s representative KYRBS database. As seen in previous studies, we have confirmed that there is an association between having same-sex intercourse and SROs. In our study, 0.5% of youths in the 12th grade, aged 17 years old, reported having same-sex intercourse (0.6% of males and 0.3% of females), and 0.5% of those youths reported having intercourse experience with both-sexes (0.6% of males and 0.3% of females). Meanwhile, from 2001 to 2009, the median of youths in the United States for students in grades 9 to 12, aged 14–17 years old, was 2.5% for same-sex intercourse and 3.3% for intercourse with both-sexes [16]. The prevalence of same-sex intercourse among youths is lower in South Korea than in western countries. However, the odds of each SRO of youths with same-sex intercourse experience were comparable between LGB youths in South Korea and in the west. Our results demonstrate that the prevalence for suicidal attempts were higher among youth with same-sex intercourse (28.4% for females, 17.0% for males) and both-sex intercourse (34.1% for females, 16.8% for males) experience than those with opposite-sex intercourse (18.0% for females, 7.4% for males) experience. The odds ratios [OR] among males with same-sex intercourse experience were 1.6 for suicidal ideation, 2.3 for plans for suicide, and 2.5 for suicidal attempts. These findings correspond to the previous meta-analytic review in the west, which reported that the risk of suicide among LGB youths was 2.9 times higher than in heterosexual youths [4, 13, 17]. In adjusted analyses, males with same- or both-sex intercourse experiences were higher in SROs compared with males who had only experienced opposite-sex intercourse. Many studies have reported higher rates of SROs among gay males when compared to lesbian females [3, 18, 19]. Based on our study, we were able to confirm that violence is a risk factor for suicide among youths with same- or both-sex intercourse. Our study shows that youths with same- or both-sex intercourse experience is far higher when combined with victims from violence. These findings are consistent with prior studies that highlight the link between homophobia and masculinity in the lives of adolescent boys. In previous studies, more than half of the gay youths surveyed reported having been verbally humiliated by their peers [20] and about 30% reported having been physically assaulted [21]. One-third of LGB youths have reported experiencing verbal abuse from their families, and 1/4 have reported being physically bullied by their peers. That is, the prevalence of victims of violence of LGB youths is similar in Asian countries when compared to western countries [8, 22]. It has been suggested that LGB youths hide their concerns about sexual orientation, feelings of isolation or helplessness, and stigmatization. This results in higher levels of stress in LGB youths than in heterosexual youths in a society that is full of negativity and discrimination towards homosexuals [13, 23–25]. In Asian society, homosexuality has been considered unnatural for ages. A social atmosphere of condemning and forbidding homosexuality has been established, leading to a stigmatization of homosexuality as a pathological disorder. In the current study, LGB youths living in Asian cities received little support from their friends, and commonly experienced teasing or were treated unfairly [22, 26]. One can imagine the difficulties that Korean LGBs may experience with such social prejudice against homosexuality. These difficulties isolate LGB youths and bring about conflict, thereby leading to violence and depression that can increase the risk of suicide [26]. Given that some LGB youths have been victims of violence, LGB youths can be considered a high-risk group for suicide [24, 27]. The results suggest that the prevention of victimization among LGB youths can decrease hopelessness and depression, perhaps leading to less suicidal attempts [7, 23]. Our results suggest that supportive and inclusive social environments for sexual orientation may lower the risk of suicide attempts among LGB youths [28, 29]. Creating policies of preventing school violence, including bullying in particular, is one of the most important protection factors for LGB youths [28, 30]. Recent research has shown that higher levels of protectiveness and supportive school climates for LGB youths reduced suicidal thoughts of LGB youths, even after controlling for confounding variables [29]. Moreover, parental support has been reported to affect the health and well-being of LGB youths [31, 32]. Health risk behaviors and subjective perception of an individual’s own health, including depression, have been shown to be negative among LGB youths who lack parental support [15, 23, 31, 33]. Also, in order to decrease SROs among youths there is a need to improve the negative impression and attitude that society displays towards homosexuals, and a need to establish anti-bullying policies towards LGB youths [28, 34]. On the same note, family-based interventions can decrease hopelessness and depression, thereby leading to less suicide attempts [35]. Countries are obligated to establish policies that reinforce the family’s role to ensure parental love and support towards children and youths [2]. This reinforcement can help create a social atmosphere that allows understanding and is receptive towards an individual’s own sexual identity and that of others. SROs among youths with same- and both-sex intercourse experience seemed to be strongly associated with exposure to violence and sexual health risk. These results may be used for programs that prevent suicide as a result of issues related to intercourse in youths and national policies that will make accessible social support systems for LGB youths. Our findings of elevated levels of SROs, STDs (including HIV infection), and being a victim of violence and/or hopelessness in youths with same- and both-sex intercourse experience, provide a public health rationale for implementing safe school programs designed to prevent violence. Awareness of the relationship between these variables and being LGB is especially important for school health programs that are funded by STD (including HIV) funding streams [13, 36]. The efforts of school health departments to reduce STD-related health risk behaviors must be implemented to decrease rates of SROs. Youths with same- and both-sex intercourse experience have a higher association with the risk of suicide than youths with opposite-sex intercourse experience. It is necessary to implement prevention programs for suicide as a result of sexual intercourse in youths and establish social support systems. Additionally, educational departments should instill acceptance of diversity and respect towards life into school systems. Schools should consider integrating specific content about LGB health risks and health disparities in training regarding violence, STDs (including HIV infection), and suicide prevention. For too many LGB and gender variant students, school violence has resulted in school failure and restricted life chances that limit vocational opportunities and undermine the victim’s human potential. Therefore, school educators must continue to advocate for these youths and implement LGB inclusive policies and programs in order to promote safe and supportive learning environments where all students are protected from sexual risk behavior. Our results serve as fundamental data demonstrating that intercourse in youths contributes to the risk of suicide. As such, this data may be used for suicide as a result of issues related to sexual intercourse prevention programs, national policies that will make accessible social support systems for LGB youths, and for comparison data on sexual behaviors among youths in Korea and other countries.

### Strengths and limitations of the study

Strengths of our study were that we were able to minimize selection bias by using a representative sampling of middle and high school students nationwide. In addition, our database included a sample size of 146,621 students, which is a large enough population. Finally, the KYRBS questionnaire used in our study demonstrated high consistency and reproducibility by showing a comparable trend of results annually. Limitations of our study should also be noted. First, our study was a cross-sectional study; therefore, a temporal relationship cannot be established between type of sexual intercourse and SROs. Future longitudinal research is needed in order to further investigate the major risk factors for suicide. Second, limitations of our study include analyzing the association between type of sexual intercourse and SROs among youths without any self-reported information on sexual orientation. This made us unable to demonstrate the prevalence of LGB youths in South Korea. In addition, we regarded youths with same-sex or both-sex intercourse experience as lesbian, gay, or bisexual. This was because we could not get information about sexual orientation on the basis of sexual identity and sexual attraction. However, in other research, the pattern of risk factors significantly associated with SROs among youths was nearly the same for type of sexual intercourse experience as it was for sexual identity [3]. Therefore, future research should use a combination of information on sexual orientation and type of sexual intercourse experience as predictors of SROs. In addition, we were not able collect information about the frequency of sexual intercourse. Future research is needed in order to determine the relation between the frequency of sexual intercourse and SROs. Third, we did not control for social factors, such as school climate or connectedness, which may have negative psychological effects on LGB youths [29]. These measures were not collected or were unavailable. LGB youths may respond to school climate in unhealthy ways (sexual risk behavior and alcohol, tobacco use) or isolate themselves. Finally, various risk factors (violence, hopelessness, health risk behavior) included in our analyses may mediate the associations between type of sexual intercourse experience and SROs. However, we cannot strictly test a major risk factor in our study because we do not know the time sequences of those risk factors in relation to the timing of type of sexual intercourse experience and SROs.

## Conclusions

Compared to youths with experience of opposite-sex intercourse, youths with same- and both-sex intercourse experience were higher in all ORs of outcomes for the suicide risk than youths with only opposite-sex intercourse experience. Youths with sexual intercourse experience showed a higher association with outcomes for the suicide risk than youths who had no intercourse experience. In the multiple logistic regression analysis, after adjusting for family structure, health risk behaviors, STDs (including HIV infection), and violence, males with same-sex or both-sex intercourse experience were higher in their odds of SROs compared with males with opposite-sex intercourse experience only. We detected that SROs in youths with same-sex or both-sex intercourse experience had strong associations with gender (males), violence, and STDs. This study provides evidence that SROs for LGB youths are related to violence and STDs, controlling for risk factors. Therefore, school educators must continue to advocate for these youths and implement LGB inclusive policies and programs in order to promote safe and supportive learning environments where all students are protected from health risk behavior.

## Abbreviations

AOR: Adjusted odds ratio
CI: Confidence interval
KYRBS: Korea youth risk behavior web-based survey
LGB: Lesbian, gay, or bisexual
OR: Odds ratio
SROs: Suicide risk outcomes
STDs: Sexually transmitted diseases
